# Protective effects of MET channels on aminoglycosides- and cisplatin-induced ototoxicity

**DOI:** 10.7150/ijms.103270

**Published:** 2025-01-13

**Authors:** Lile Ouyang, Lu Ma, Yong Feng

**Affiliations:** 1 Department of Otorhinolaryngology, The Affiliated Changsha Central Hospital, Hengyang Medical School, University of South China, Changsha 410028, China.; 2 Institute of Otorhinolaryngology, Head and Neck Surgery, University of South China, Changsha 410028, China.; 3 MOE Key Lab of Rare Pediatric Diseases & Institute for Future Sciences, University of South China, Changsha 410008, China.; 4 Institute of Cytology and Genetics, Hengyang Medical School, University of South China, Hengyang 421000, China.

## Abstract

Aminoglycosides and cisplatin drugs are extensively utilized for their high efficacy in treating various conditions in the clinic, however, their ototoxic side effects warrant significant attention. These drugs could penetrate the inner ear via specific channels or transporters, which not only affect the survival of hair cells but also induce the overproduction of reactive oxygen species. Currently, scientific research mainly addresses this issue through the downstream intervention of reactive oxygen species. However, recent studies have revealed that directly reducing the uptake of these drugs by hair cells can effectively avoid initial damage. In particular, the interactions between drugs and hair cells, as well as the specific functions of relevant channels and transporters, can be explored in detail through the use of molecular dynamics simulations. The swift advancement in the field of structural biology has shed light on the structural functions of various channels and transporters closely related to drug absorption, such as electromechanical transduction channels (MET) and organic cation transporter-2, etc., providing theoretical basis and potential targets for novel ear protection strategies. It is, therefore, imperative to investigate the regulatory role of the MET channel in the up-taking of ototoxic drugs, serving as a pivotal point for the development of preventative and therapeutic approaches. This review aims to highlight the mechanism of inhibition of ototoxic substances absorption by auditory hair cells, explore how to develop novel ear protection methods by targeting these channels and transporters, and provide a new perspective and strategy for addressing drug-induced ototoxicity. The approach to protecting hair cells by targeting these channels and transporters not only broadens our understanding of the underlying mechanisms of ototoxicity, but could also spur further research and progress in the field of auditory protection.

## Introduction

### General concept of ototoxicity

Ototoxicity is drug-induced inner ear tissue damage 1, primarily caused by the cytotoxic effects of certain medications 2. Aminoglycoside antibiotics, known for their significant therapeutic efficacy, are widely used to treat multidrug-resistant gram-negative bacterial infections. However, there is a concurrent significant risk of ototoxicity, which can cause irreversible damage to cochlear hair cells 3. Additionally, cisplatin, a commonly used antineoplastic drug, exhibits particularly severe ototoxicity. It can lead to apoptosis 4 and necrosis 5 in hair cells through DNA adduct formation 6, mitochondrial dysfunction 7, oxidative stress 8, and inflammatory responses 9. This not only limits the use of cisplatin but also means that current treatments for cisplatin-induced ototoxicity mainly rely on prosthetic devices, which have limited effectiveness and do not fundamentally address the issue of inner ear damage 10. Such drug-induced hearing loss severely affects the quality of life of patients, increases their communication barriers, and may lead to increased suffering in their work and family environments, potentially even increasing their risk of depression or dementia 11. Therefore, developing novel otoprotective methods against drug-induced ototoxicity is crucial and urgent.

### Aminoglycoside ototoxicity

Aminoglycoside drugs are an antibiotic for neonatal sepsis 12 and gram-negative bacterial infection 13, and play an important role in antibiotic treatment. Due to their low cost and low risk of antibiotic resistance, they are considered the drug of choice in many cases, especially in developing countries. However, in clinical applications, these drugs have been found to have toxic effects on the kidneys and inner ear. Although kidney damage is usually reversible 14, approximately 20% of patients treated with aminoglycosides develop long-term permanent hearing loss 15, 16. Therefore, aminoglycoside-induced hearing loss is one of the most common hearing loss 17. Depending on different dosing regimens and the sensitivity of audiological tests, it is estimated that the proportion of hearing loss that occurs after receiving aminoglycoside therapy varies widely, but may be as high as 50% in some cases. In addition, vestibular toxicity may occur in up to 60% of cases during treatment 18.

### Cisplatin ototoxicity

Cisplatin is a widely used antitumor drug 19. However, it has a wide range of toxicity, affecting the gastrointestinal tract, blood system, kidney, auditory system 20, neurons, and also inducing DNA damage 21. Among them, cisplatin-induced ototoxicity is one of the most serious adverse reactions during the administration of cisplatin, which may lead to irreversible, progressive, bilateral and cumulative hearing damage, thus limiting the clinical application of cisplatin 22. Although the research on cisplatin damage to cochlea has been widely carried out in various fields in recent years, there are relatively few reports on the mechanism of *in vitro* toxicity induced by cisplatin 23. Vestibular hair cells in the extrastriola area are more susceptible to cisplatin than hair cells in the striola area 19. While it is well established that cisplatin can damage hair cells in the inner ear, scientists have observed that the relationship between cisplatin dosage and vestibular hair cell loss follows a U-shaped curve rather than a linear pattern 24. They treated rat vestibular explants (utricle and saccule ex vivo cultures) with cisplatin at concentrations ranging from 10 to 1000 μM. As the concentration of cisplatin increased from 10 μM to 50 μM, the number of vestibular hair cells decreased. Conversely, when the cisplatin dose was increased from 100 μM to 1000 μM, the number of vestibular hair cells began to increase. When treated with 1000 μM cisplatin, nearly all hair cells in the culture survived, with the greatest hair cell loss occurring within the 50-100 μM range 24. In contrast to the phenomenon where hair cells or ganglion neurons suffer little damage after treatment with 1000 μM cisplatin, most vestibular nerve fibers and peripheral nerve terminals remain absent after treatment with high doses of cisplatin. These results indicate that, compared to hair cells or neuronal bodies, peripheral nerve fibers and terminals are more sensitive to cisplatin-induced damage. Therefore, when establishing a cisplatin damage model in experiments for the study of ototoxicity protective drugs, controlling the concentration of cisplatin around 50-100 μM will more accurately assess the effectiveness of the ototoxicity protective drugs if the intention is to evaluate the damage to vestibular hair cells. The majority of existing research on cisplatin ototoxicity and ear protective drugs, including many of the related drugs and compound experiments mentioned later in this paper, use cisplatin treatment concentrations that are close to this range.

## Exploring the pathway and mechanism of cisplatin- or aminoglycosides-induced ototoxicity

### Cellular uptake and ototoxic mechanisms of aminoglycosides and cisplatin

When discussing the mechanism of ototoxicity of cisplatin and aminoglycoside drugs, it should not be ignored that they cause effects at the cellular level. Cisplatin mainly exhibits its toxic effects by inducing apoptosis, while aminoglycosides trigger both apoptosis and necrosis 25. The implementation of these mechanisms occurs in a significant microenvironment of the inner ear, where the ion composition and fluid volume of the endolymphatic environment (i.e., low Na^+^, high K^+^) of the cochlea are significantly different from perilymph environment (i.e., high Na^+^, low K^+^) and larger fluid volume 26. The pathway by which cisplatin and aminoglycoside antibiotics enter the cells can be roughly divided into two steps and the pathway from the blood to the inner ear can be divided into two key steps. As the first step, studies have shown that ototoxic drugs, such as aminoglycoside antibiotics and cisplatin, exit the Blood-Labyrinth Barrier (BLB) into the stria vascularis and subsequently enter the endolymph-filled cochlear duct. The BLB is a unique barrier system consisting of endothelial cells joined by tight junction proteins, which prevent macromolecules and blood cells from passively exiting capillaries into cochlear tissues. However, small molecules such as cisplatin and aminoglycoside antibiotics can cross this barrier 27 This entry exposes hair cells to these ototoxic drugs, allowing them to enter the hair cells through their apical membrane, leading to ototoxic effects 28. For aminoglycoside antibiotics and cisplatin, while their route of entry is understood, the molecular mechanism by which they enter the endolymph-filled the cochlear duct is less understood. Previous studies have suggested that these drugs may enter through transient receptor potential ankyrin 1 (TRPA1) channels 29. In addition, studies have shown that inflammation and noise exposure can activate TRP channels 30, thereby increasing the absorption rate of aminoglycosides. Although endocytosis is another known pathway into hair cells, blocking this pathway or interfering with the intracellular transport of aminoglycosides has not been effective in preventing hair cell damage 18, 31. The transport process of cisplatin is relatively complex, and it can directly cross the cell membrane and enter the cell through passive diffusion. In particular, in marginal cells, cisplatin mainly uses Organic Cation Transporter-2 (OCT-2) and Copper transporter-1 (CTR1) to enter the endolymph 30, 32. In the hair cell stage, cisplatin and aminoglycosides enter through specific cell membrane channels and accumulate in the cell through endocytosis, leading to the ultimate manifestation of cytotoxicity.

### Mechanism of aminoglycoside ototoxicity

The mechanism of ototoxicity induced by aminoglycoside mainly involves two aspects. First, the accumulation of these drugs in the inner ear can cause significant damage to hair cells. Aminoglycoside drugs affect ATP synthesis by increasing oxidative stress within cells and disrupting mitochondrial structures, leading to reduced ATP synthesis, thereby inducing hair cell dysfunction and death 33. This process directly affects the normal transmission of auditory signals and may lead to hearing loss 2. Furthermore, aminoglycoside antibiotics may also have toxic effects on the auditory nerve itself, interfering with its conduction function and exacerbating hearing problems. Regarding how aminoglycosides enter hair cells, studies have shown that the MET channel is the main entrance, through which aminoglycosides accumulate in the cell and cause cell death. In addition, TRPV1, TRPV4 and TRPA1 channels located in the apical membrane or basolateral membrane of cochlear hair cells are also involved in the transport process of aminoglycosides 27, 34, which also provides a new research direction for further exploring how to reduce the ototoxicity caused by aminoglycosides.

### Mechanism of cisplatin-induced ototoxicity

The mechanism of cisplatin-induced ototoxicity mainly includes three main aspects. First of all, the direct damage of cisplatin to the auditory nerve may be closely related to oxidative stress and cell apoptosis, which leads to nerve conduction disorders and hearing loss. Secondly, the accumulation of cisplatin in the inner ear can affect the normal function of inner ear cells, and the oxidative damage and apoptosis of these cells further impair hearing 35. Finally, cisplatin disrupts the function of the cochlea, activating transcription factors and signal converters in cochlea cells, such as TRPV1 and NADPH oxidase 3, which increases cochlea edema, inflammation, and cell damage, leading to hearing loss. During this process, high levels of reactive oxygen species are the main inducer of cell death 36. By using inhibitors and potential blockers of the main channels on hair cells (including OCT2, CTR1, MET, and TRPV1), the cellular uptake of cisplatin can be disrupted.

## The regulatory role of MET channels in the absorption of ototoxic drugs

### Composition and mechanism of MET channel

**Figure 1:**
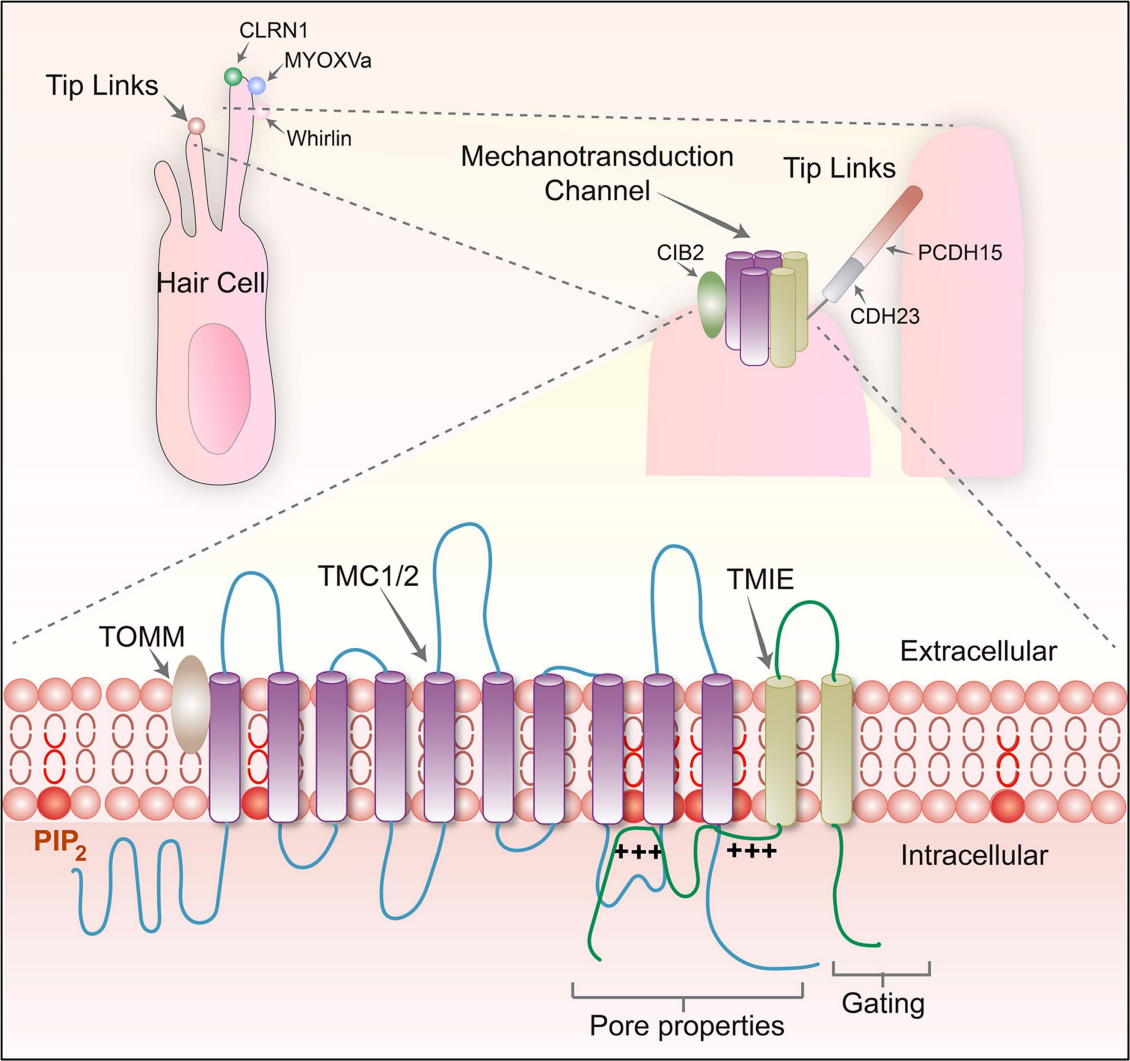
Diagram of the structure and composition of the MET channel complex protein, modified from Cunningham *et al.* (2020) 23.

When discussing the basic mechanism of the auditory system, the role of MET channels located on the cochlear hair cells cannot be ignored. These channels convert mechanical acoustic signals into electrical signals that the nervous system can recognize. In short, when sound waves travel through the cochlea, they trigger the movement of cilia on the surface of the hair cell, triggering the opening of the MET channel, which then encourages the depolarizing influx of ions into hair cells and the generation of electrical signals. This process of depolarization, within the hair cells, leads to the inward flow of calcium ions and other ions, thereby producing electrical signals that can be detected and processed by the auditory nervous system. The MET channel is composed of several key proteins, including protocadherin 15 (PCDH15), cadherin 23 (CDH23), and lipoma HMGIC fusion partner-like 5 (LHFPL5). Together, these molecules build an elaborate mechanism. In addition, transmembrane inner ear (TMIE), transmembrane channel-like 1/2 (TMC1/2) and calcium and integrin-binding family member 2 (CIB2) also contribute to the function of this complex (Figure 1). Understanding the normal function of the MET channel is critical to shedding light on auditory perceptual processes. The activation of the MET channel is not only related to the change of calcium ion concentration in hair cells, but it has also been demonstrated in a culture study based on a tumor cell line to affect various biological behaviors of cells, such as growth, proliferation and migration 37. The MET channel also hosts the delivery of specific drugs, particularly aminoglycoside antibiotics 38-40. These drugs enter the endolymphatic system through the BLB 41 and then cross the apical membrane of hair cells to enter the inner ear hair cells 13. Because aminoglycosides are polar molecules, they cannot travel directly through the lipid-containing membranes of hair cells. However, it is possible through the MET channel, the transient receptor potential cation channel subfamily A member 1 (TRPA1) channel, the transient receptor potential vanillin 1 (TRPV1) channel, the P2X channel, or the ion channel containing piezo2 transmembrane ion channels 18, 39 or enter the cell interior through endocytosis. Therefore, the MET channel is not only a central element in understanding the auditory conduction process, but also a key to studying treatment strategies for deafness and related diseases. The in-depth study of the molecular structure and function of these channels can provide a theoretical basis for the design of novel hearing restoration methods and even the development of more accurate drug delivery systems.

### The research model related to the MET channel and the current research progress of related drugs

#### Research models and drugs (Table 1)

##### Research model and progress related to UoS-7692

**Figure 2:**
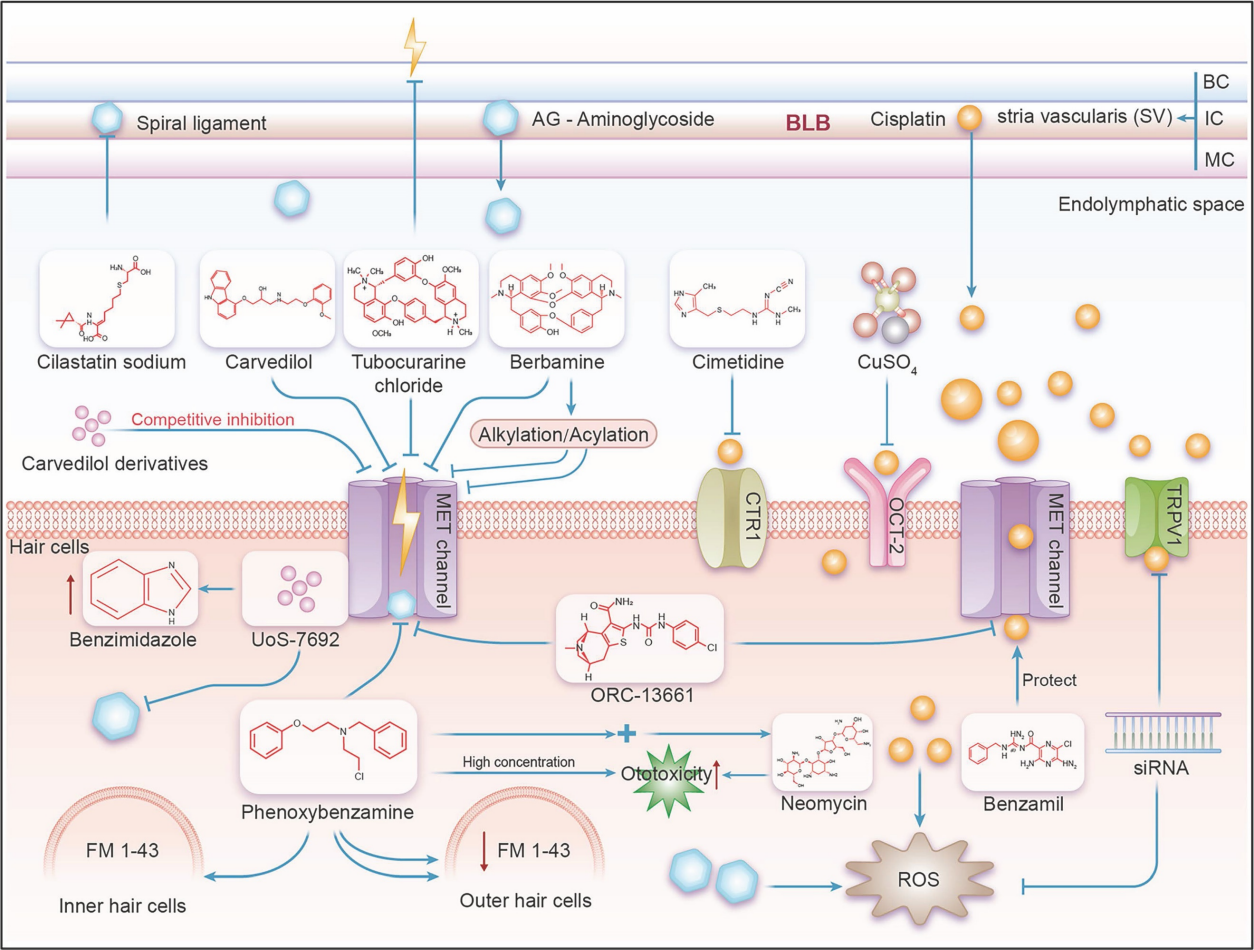
** In mammals, a schematic diagram illustrating the mechanism by which the MET channel affects aminoglycoside- and cisplatin-induced ototoxicity.** BC=Basal Cell, IC=Intermediate Cell, MC=Marginal Cell. As shown in this figure, ototoxic drugs such as aminoglycosides and cisplatin enter the stria vascularis from the BLB, and then proceed into the endolymph, exposing hair cells to these ototoxic substances. This allows the drugs to enter the hair cells through their apical membrane, leading to ototoxic effects. Various effective compounds, including inhibitors and potential channel blockers, act on the main channels of the hair cells (including OCT2, CTR1, MET, and TRPV1), interfering with the cell's uptake of ototoxic drugs like aminoglycosides and cisplatin, thereby achieving the effect of ototoxic protection.

In the realm of contemporary otological research, addressing the issue of ototoxicity induced by aminoglycoside antibiotics has prompted a research consortium to engage in extensive compound screenings utilizing zebrafish and murine models 42. A seminal breakthrough within this domain was the identification of a novel small molecule, designated UoS-7692, which exhibited remarkable protective properties (Figure 2). During the initial phase of the investigation, the research team scrutinized over 10,000 compounds (specifically, 10,240) in zebrafish larvae, meticulously selecting those demonstrating resilience against the toxic effects of neomycin and gentamicin. Advancing their inquiry, the researchers conducted a meticulous screening process of 64 samples within mouse cochlear cultures. This endeavor culminated in the discovery of eight compounds that manifested protective effects for outer hair cells (OHCs), without inflicting supplementary structural damage to the hair bundles. The primary mechanism of action of these protective compounds involves the blockade of the MET channel in OHCs, a pivotal route for gentamicin penetration. UoS-7692 distinguished itself by significantly forestalling the accumulation of gentamicin within OHCs and evincing protective efficacy against other aminoglycoside antibiotics, including kanamycin and tobramycin. An in-depth dissection of UoS-7692 revealed that its protective mechanism is devoid of any impact on the bactericidal potency of gentamicin. Furthermore, in functional assays utilizing the zebrafish lateral line system, UoS-7692 was demonstrated to retain its biological activity after neomycin exposure. In subsequent *in vivo* experiments, the compound was administered to mice via transtympanic injection, yielding outcomes that underscored its efficacy in safeguarding the OHCs from furosine/kanamycin-induced injury and in preserving auditory function to a notable extent. Collectively, these experimental series substantiated the MET channel in OHCs as a viable target for countering aminoglycoside-induced ototoxicity, concurrently furnishing pivotal chemical insights instrumental for the conceptualization and development of forthcoming cochlear protective agents 43. The progression of this research is anticipated to catalyze the evolution of a new cadre of auricular therapeutics, designed to mitigate the risks of intracochlear toxicity engendered by aminoglycoside antibiotics, thereby enhancing the global patient populace's quality of life and offering a constellation of targeted compounds that will inform the architecture of next-generation cochlear protective devices.

##### Research model and progress related to d-Tubocurarine

Within the context of the zebrafish lateral line system, investigations have revealed that d-Tubocurarine and berbamine are capable of exerting a complete protective effect on hair cells within zebrafish lateral line organs, shielding them from the deleterious impacts of aminoglycoside antibiotics. Furthermore, it has been observed that the modulatory influence of d-Tubocurarine and berbamine on the MET channels is associated with their pharmacological interaction with nicoxamide (Figure 2). The research team has also conceptualized a potential strategy to augment the MET channel's selectivity. This involves structural modifications to d-Tubocurarine aimed at elevating its MET channel affinity while concurrently neutralizing its cellular entry capacity 44. These collective discoveries pave the way for the advancement of investigative endeavors and the formulation of protective strategies targeted at averting cochlear damage.

#### Research model and progress related to berbamine

Berbamine analogs have been found to protect auditory hair cells from aminoglycosides by blocking mechanoelectrical transduction (MET) channels through the zebrafish lateral line system 44. These analogs specifically impede the entry of aminoglycosides into hair cells by engaging with the MET channels, thereby altering the channel's conformation or obstructing its open state (Figure 2). Such intervention is instrumental in safeguarding hair cells from aminoglycoside-induced injury. Additionally, it has been discovered that certain berbamine analogs possess multimodal protective mechanisms, suggesting that they may exert their effects through pathways beyond the blockade of MET channels. Current research has demonstrated that acylated and alkylated derivatives of berbamine exhibit enhanced protective efficacy compared to the parent compound, implying that these structural modifications contribute to the mitigation of hair cell damage 45. These multifaceted protective mechanisms engender these compounds as promising candidates for therapeutic development, given their potential to modulate multiple targets concurrently and curb hair cell demise 45.

##### Research model and progress related to FM1-43 derivatives

Fluorescent dye FM1-43 and its derivatives are potential blockers of the MET channels in auditory hair cells 46. These dyes are also commonly used to detect the activity of MET channels 39. To be specific, In the realm of murine models, scientists have investigated the hypothesis that systemically administered, fluorescently-labeled gentamicin gains primary access to mouse hair cells through the MET channels. The MET channels are integral to the functionality of sensory hair cells, with transmembrane channel-like proteins 1 (TMC1) and TMC2 being postulated as components of these sensory MET channels 47. Employing mouse models deficient in *Tmc1* (*Tmc1*^Δ/Δ^), *Tmc2* (*Tmc2*^Δ/Δ^), and a model with TMC1 tagged with a red fluorescent protein (*Tmc1-mCherry*), the experiments have unveiled that in *Tmc1*^Δ/Δ^;*Tmc2*^Δ/Δ^ mice, the uptake of gentamicin Texas Red (GTTR) is markedly reduced compared to that in wild-type hair cells 47. Furthermore, as the sensory MET function in mouse hair cells matures, there is a gradual enhancement in GTTR fluorescence intensity, whereas in hair cells lacking sensory MET, the GTTR fluorescence intensity remained low 47. It was also observed that even without apparent interruption of endocytosis at the apical surface of the hair cells, *Tmc1*^Δ/Δ^;*Tmc*2^Δ/Δ^ hair cells exhibited low GTTR fluorescence intensity. Additionally, it was found that only those hair cells that significantly took up systemic GTTR also took up FM1-43, a dye used to detect MET channel activity 39 (Figure 2). Therefore, in future studies related to MET channels, it can be fully utilized as a real-time measurement tool to verify the accuracy, thereby promoting the development of related ototoxicity protective drugs. Using the mouse cochlear culture model as a measurement tool, some studies have assessed the function of MET channels by measuring the uptake of the FM1-43 fluorescent dye. The results showed that when MET channels were exposed to aminoglycoside drugs and FM1-43 fluorescent dye, the drug uptake led to damage to the MET channels and cell death. However, when hair cells were pretreated with phenoxybenzamine (A known antagonist of alpha 1 (α1) and alpha 2 (α2) adrenergic receptors, which will be discussed in detail in subsequent sections), the uptake of FM1-43 fluorescent dye was significantly reduced, thus protecting the hair cells from the toxicity of aminoglycoside drugs. This suggests that phenoxybenzamine may have the potential to protect hair cells from the toxicity of aminoglycoside drugs 48. Other studies, through mouse and zebrafish models, have found that the lipophilic tail and the positively charged head of FM1-43 are both required for effective blocking of the MET channel, and relying solely on one part is insufficient. Through testing a series of structurally modified FM1-43 derivatives, it was found that increasing the lipophilicity/bulkiness of the tail, reducing the number of positive charges in the head from two to one, or increasing the distance between the two charged head groups, all enhanced the blockage of the MET channel 49. The blocking effect of FM1-43 derivatives on the MET channel and their loading efficiency in hair cells can be modulated through chemical modification. Certain FM1-43 derivatives have the potential to protect hair cells from the ototoxicity of aminoglycoside antibiotics at low concentrations, but this protective effect may be accompanied by some degree of disruption of the hair bundle structure 49. Thus, the study of FM1-43 and its derivatives has revealed that their chemical structure is crucial for interaction with the MET channel and how their efficacy can be modulated through chemical modification. Although some derivatives show potential in protecting hair cells from damage caused by aminoglycoside antibiotics, future research can build on this foundation to determine their specific mechanisms of action and application potential, advancing the development of ototoxicity protective drugs in new fields.

##### Research model and progress related to carvedilol

Investigators have recently explored a novel derivative of carvedilol, demonstrating its capacity to safeguard auditory sensory hair cells from aminoglycoside-induced injury by inhibiting mechanical electrotransduction channels 35 (Figure 2). Carvedilol, a non-selective antagonist of α1 and β-adrenergic receptors, is widely utilized in clinical settings for the management of hypertension, angina, and chronic heart failure. The carvedilol derivatives, a collection of compounds derived from the chemical modification of the parent molecule, have been synthesized and characterized 50. A comprehensive experimental approach was employed to ascertain the protective efficacy of these novel compounds. Initially, the researchers applied electrophysiological methods to scrutinize the impact of these derivatives on MET channels. They delineated the concentration-response profiles for both carvedilol and its derivatives, subsequently employing mathematical modeling to elucidate the kinetics of channel penetration and blockade 35. Subsequently, *in vitro* assays were conducted using cochlear cells from mice to evaluate the influence of these derivatives on aminoglycoside uptake and subsequent cellular damage. Cells were cultured in media supplemented with carvedilol, its derivatives, or control substances, followed by the introduction of aminoglycosides. Cellular fluorescence was monitored and quantitatively assessed via *in vivo* imaging techniques. Notably, in the context of mouse cochlear cell cultures, carvedilol was observed to fully shield hair cells from gentamicin-induced damage at concentrations of 10 μM and 20 μM, specifically when exposed to 5 μM gentamicin. However, at concentrations exceeding 30 μM, cytotoxic effects were observed. The experimental findings collectively indicate that carvedilol functions as a high-affinity, permeable, and reversible MET channel inhibitor. The protective mechanism of carvedilol is contingent upon the presence of carbazole groups, and its interaction with MET channels is independent of hydroxyl groups. Ultimately, the research revealed that the newly synthesized carvedilol derivatives were efficacious in preserving sensory hair cells from the detrimental effects of aminoglycosides, thereby presenting themselves as promising candidates for the development of innovative cochlear protective agents 35. The elucidation of a structure-activity relationship through the study of these derivatives has facilitated the design and optimization of novel compounds endowed with superior pharmacokinetic profiles and diminished toxicity, thereby offering a viable strategy for mitigating ototoxicity.

##### Research model and progress related to ORC-13661

Research on ORC-13661 has revealed its effects on the outer hair cells of the mouse cochlea, where it was discovered that mutations in the TMC1 and TMC2 genes can affect the calcium sensitivity of the mechanoelectrical transduction (MET) channel and the blocking action of dihydrostreptomycin 34. By examining the cochlear outer hair cells during the early postnatal development phase in mice, it has been found that the TMC2 gene can alter the permeability characteristics of the MET channel 51. Studies on the relationship between inner ear hair cells and membrane protein structures have unveiled the structural relationship between the TMC1 protein and the TMEM16 protein, that is, within the MET channels, TMEM16 family proteins share structural and functional similarities with TMC1 52. TMC1 is considered a key component of the MET channel, and TMEM16A and TMEM16F are homologous to TMC1 in both sequence and structure, as they are all calcium-activated ion channels 53. The research outcomes demonstrate that ORC-13661 is a permeant blocker capable of binding to the MET channel and inhibiting its function (Figure 2). The specific mechanisms are as follows: 1. Binding of ORC-13661 to the MET channel: Experimental results indicate that ORC-13661 binds to the MET channel and interacts with the cation binding sites within the channel. This binding is negatively cooperative, meaning that two molecules of ORC-13661 bind to a single channel pore. This binding mode is similar to how other cationic compounds interact with the MET channel. 2. Blocking effect of ORC-13661: The blocking effect of ORC-13661 is related to the cell's membrane potential. Under negative potentials, ORC-13661 can effectively inhibit the function of the MET channel, while under positive potentials, the blocking effect is reduced. This suggests that the blocking action of ORC-13661 is associated with the electrochemical gradient across the cell membrane. 3. Kinetic characteristics of ORC-13661: Experimental data show that the blocking action of ORC-13661 has certain kinetic characteristics. It can block the channel's function before the channel opens, indicating that it is a closed-channel blocker. Moreover, the blocking effect of ORC-13661 increases with concentration, but within a certain concentration range, the blocking effect weakens as the cell becomes hyperpolarized 54. Recent studies have also found that the protective effect of ORC-13661 may require multiple doses to achieve optimal results 55. These findings on ORC-13661 provide an important theoretical foundation for the development of new hearing protection drugs.

##### Research model and progress related to Phenoxybenzamine and benzamil

Phenoxybenzamine is a well-known antagonist of alpha 1 (α1) and alpha 2 (α2) adrenergic receptors 56. Phenoxybenzamine exhibits a slower onset and longer duration of action compared to other α-adrenergic receptor blockers at the α1-AN and α2-A receptors. It is utilized to reduce vasoconstriction induced by epinephrine and norepinephrine. Additionally, phenoxybenzamine acts as a reversible blocker of MET channels (Figure 2). Studies using the zebrafish system have revealed that it significantly decreases the rapid uptake of FM 1-43 in outer hair cells (OHCs) rather than inner hair cells (IHCs) 57. When co-applied with FM 1-43, phenoxybenzamine does not significantly affect the uptake of FM 1-43. However, pre-incubation with phenoxybenzamine for 30 or 60 minutes followed by treatment with FM 1-43 markedly reduces its uptake. This pre-treatment decreases the uptake of FM 1-43 to 28% in OHCs and 60% in IHCs compared to the control group, and the effect is reversible 57. Phenoxybenzamine demonstrates a protective effect on both OHCs and IHCs, reducing hair cell death caused by neomycin. At a concentration of 200 μM, phenoxybenzamine exhibits some toxicity to OHCs, and when used in combination with neomycin, its toxicity to OHCs is enhanced, showing cumulative toxicity 57. This suggests that the blocking effect of phenoxybenzamine on FM 1-43 uptake varies between the two types of hair cells, indicating differences in the structure or regulation of MET channels between them. Therefore, at appropriate concentrations, phenoxybenzamine can reduce aminoglycoside (AG) uptake by blocking the open state of MET channels, thereby protecting hair cells from AG toxicity 57. Regarding cisplatin-induced ototoxicity, recent studies have also confirmed that benzamil exhibits significant protective effects on cochlear explant hair cells against cisplatin toxicity 58. Further investigation into the roles of phenoxybenzamine and benzamil in different hair cell types may aid in understanding the distinct regulatory mechanisms of MET channels and provide information for the development of more effective and safer ototoxicity-protective agents.

#### Molecular mechanisms related to MET channels

In the exploration of genetic mechanisms underlying deafness, a spectrum of proteins, in addition to TMC1 and TMC2, have been identified as integral components of the MET channel in hair cells. Beyond these two, approximately a dozen proteins collaborate to constitute the MET channel, and the study of these constituents offers profound insights into the intricate molecular underpinnings of this phenomenon. In recent years, a novel integrative model has emerged, synthesizing previously discordant perspectives on the MET channels' role in hair cell mechanosensation. This model, grounded in the latest empirical data and scholarly research, posits that the MET channel interfaces with the cytoskeleton through CIB/ankyrin, rather than being directly affixed to the apical link. Upon hair bundle deflection, the apical binding force dilates the shorter stereociliary apical membrane, thereby instigating membrane stretching and spring forces within the CIB/ankyrin cytoskeleton anchor, which in concert, triggers the opening of the TMC1/2 channel. In this framework, the role of the tip link is to convey localized membrane tension in proximity to the TMC/CIB/ankyrin complex, rather than being directly integrated into it. Furthermore, the model delineates the formation of complexes by the LHFPL5 protein and the PCDH15 dimer, with TMIE potentially serving as an accessory subunit of the TMC1/2 complex 23, modulating channel gating and pore characteristics or even facilitating pore formation. This model aligns with current experimental evidence, yet necessitates further validation through structural and functional data analysis and refinement. In a study of interactions with the MET channel, benzimidazole group-containing compounds exhibit enhanced affinity for the MET channel, with compounds UoS-7692, UoS-8052, UoS-3607, and UoS-3606 achieving over 70% MET current blockade at 50 μM concentrations. In contrast, UoS-7691 demonstrates a reduced efficacy, accounting for only 20% of the blockage. UoS-7691, characterized by its least lipophilic properties due to a furan ring, stands apart from the more lipophilic compounds, which may account for its diminished effectiveness 59. This also reflects that the activity of the MET channel may be regulated to some extent by changing the lipophilic ability of the compounds that affect the MET channel. Recent discoveries propose that the ion conduction pathway of the MET channel subunit TMC1 might be a groove close to the lipid bilayer. Consequently, the apparent influence of lipophilic aromatic moieties in augmenting the capture has garnered significant interest 25, 34. This implies that lipophilicity may foster the compound's interaction with the negatively charged aminoglycoside binding sites situated deep within the conduction pathway. Such findings pave the way for novel avenues in the investigation of MET channel mechanisms and the development of ototoxicity-mitigating drugs for aminoglycosides.

## Current gaps in the knowledge base on ototoxicity induced by MET channel mechanisms

In the realm of ototoxicity research, there remain significant gaps in our comprehension of the operational mechanisms of the MET channel. The reconstitution of the MET mechanism, whether in heterologous cell systems or utilizing artificial lipid bilayers, represents a pivotal step in advancing our knowledge. These studies expand our understanding of the molecular architecture of the MET complex, offering novel investigative tools to explore force-mediated gating mechanisms.

Future endeavors will concentrate on elucidating the atomic-level structure of TMC1 and TMC2 proteins within the MET channel, thereby revealing the complete configuration of the entire MET complex 60. It is noteworthy that the identification of TMC1, originating from studies on genes associated with nonsyndromic sensorineural hearing loss 19, signifies a landmark achievement. This research not only enhances our grasp of the molecular etiology of deafness but also informs the development of prospective therapeutic interventions. Additionally, other studies have delineated that the static stereocilia bundles of outer hair cells exhibit a distinct orientation, referred to as the planar cell polarity (PCP) of hair cells. Incoming auditory signals to the inner ear have the potential to influence these resting cilia. Nonetheless, research about the role of PCP in ototoxicity and its implications for the MET channel's gating dynamics is limited, highlighting an area ripe for scientific inquiry and breakthrough.

## Clinical dilemmas and potential solutions and prospects

When discussing the impact of ototoxic substances on human health and strategies for their prevention and treatment, we are confronted with several key scientific questions. Initially, elucidating the exact molecular mechanisms that enable specific substances to traverse the BLB and influence cochlear cells is essential. This necessitates an examination of how cochlear cells react to these exogenous toxic stimuli and the disclosure of the underlying molecular regulatory dynamics. Moreover, discerning the precise molecular architecture of the MET channel complex will facilitate the crafting of small molecule drugs capable of efficaciously modulating its function. Such targeted interventions could foster the genesis of innovative protective measures, particularly for individuals at elevated risk due to the administration of aminoglycoside antibiotics and cisplatin-based chemotherapy agents. Furthermore, when contemplating the clinical deployment of novel interventions, the safety and efficacy of MET channel antagonists must undergo rigorous evaluation, with a focus on potential adverse effects and complications. To achieve the aforementioned points, we need to have a correct understanding of the potential and limitations of MET channel-blocking compounds and drugs. Although various drugs and compounds have been listed that can target the MET channel to alleviate ototoxicity caused by cisplatin or aminoglycosides, most of these compounds, such as d-Tubocurarine, Phenoxybenzamine, and Carvedilol, not only fail to provide ear protection at high concentrations but also enhance ototoxicity, with a narrow effective dose window limiting the practical clinical application of many MET channel blockers. Moreover, some MET channel blockers may reduce the antibacterial activity of aminoglycosides or the antitumor activity of cisplatin itself while alleviating ototoxicity, as is the case with FDA-approved Sodium Thiosulfate (Pedmark), which reduces the activity of cisplatin antitumor chemotherapy while providing protective effects 61, 62, limiting its broad clinical application in this regard. This is also a point that deserves attention and consideration. However, some non-mechanotransduction channel blockers, such as N-acetylcysteine 23, 63 and salicylates 64, 65, provide ear protection by enhancing the production of endogenous antioxidants without affecting the antitumor activity of cisplatin, which is an advantage of non-MET channel-blocking ear protection drugs. But compounds such as ORC-13661 and UoS-7692 have also been shown to provide protection against ototoxicity without affecting the antitumor and antibacterial effects of cisplatin and aminoglycosides themselves 43, 53. Therefore, these may have greater potential among MET channel blockers for clinical use. To achieve a level suitable for clinical application, further in-depth research is required, particularly conducting clinical trials related to hearing loss for drugs that have shown otoprotective effects in animal models. Looking at the field of ototoxicity protective drugs as a whole, drugs such as vitamin E, alpha-lipoic acid, *Ginkgo biloba*, berberine, and curcumin have demonstrated otoprotective effects in several animal models, but not of them passed the clinical trials against aminoglycoside induced hearing loss. Among MET channel blockers, only a few, like ORC-13661, have been attempted in clinical trials. Therefore, there are still many directions and drugs that we can explore in depth for future research. As per the clinical data presented in ARO 2024, currently, Glutathion peroxidase activator called SPI-1005 or ebselen is in clinical phase 2b study, enrolling 60 adult cystic fibrosis patients, who were receiving intravenous (IV) tobramycin. Other promising drugs can also be progressively advanced step by step, similar to the experiments conducted on the glutathione peroxidase activator ebselen. Concurrently, cellular damage caused by ototoxic substances typically involves many complex mechanisms and molecular pathways, requiring meticulous characterization and understanding to identify breakthroughs for new ototoxic drug protectants. As mentioned earlier, aminoglycoside drugs produce reactive oxygen species by damaging mitochondria, leading to hair cell death and hearing loss; however, recent studies have found that activating mitophagy to clear damaged mitochondria can alleviate aminoglycoside-induced hearing loss, providing a new therapeutic strategy for preventing hearing loss induced by aminoglycoside antibiotics 66. In the future, a new perspective on the prevention of aminoglycoside antibiotic-induced hearing loss needs to be explored from the MET channel and its related mechanisms. The points worth exploring in the MET channel have also been partially described. Prior research has indicated that the ectopic expression of Atoh1 and Tbx2 can foster the *in situ* regeneration of cochlear hair cells. Despite morphological and transcriptomic similarities to native hair cells, regenerated inner hair cells have been observed to fall short in restoring auditory function, potentially due to the absence of MET channel currents 67. In the realm of inflammatory responses, the presence of certain biomarkers may intensify the impact of ototoxic agents; thus, pinpointing these specific thresholds and their expression consistency across various inflammation types, be it bacterial, viral, drug-induced, or attributable to other factors, is imperative for mapping out prevention and control strategies. While cellular damage incited by ototoxic agents typically involves a confluence of complex molecular pathways, it is also imperative to investigate compounds with multimodal protective effects. Such agents may offer more holistic safeguarding by engaging multiple targets and curtailing hair cell demise. Exploring whether inflammatory responses triggered by diverse sources can amplify the severity of ototoxicity 20 and collectively influence the MET channel's functionality is vital for a comprehensive understanding of these pathophysiological processes and for devising efficacious interventions. Additional studies have posited the hypothesis that alternative splicing of the *Cdh23* gene modulates the hair cell's apical connections and MET channel, which could have profound implications for hair cell functionality and the amelioration of hearing loss 48. Recent studies have focused on interventions targeting the GPx4-mediated iron drop and other pathways to alleviate cisplatin-induced ototoxicity, suggesting that interventions targeting the MET channel and ferroptosis pathways may provide new therapeutic strategies for treating cisplatin-related ototoxicity 68. The MET channel is crucial for hearing, and complete blockade of the MET channel can lead to total hearing loss 69. It is also essential to address how to target the MET channel to prevent ototoxic drug-induced hearing loss without impairing hearing due to the blockade of the MET channel. Several potential solutions may include: 1. Selective inhibitors: Developing selective MET inhibitors that block specific functions of the MET channel or modulate key associated proteins, such as TMIE and LHFPL5, without completely shutting down the channel. This approach could reduce damage from ototoxic drugs while preserving other essential functions of the MET channel. 2. Time-controlled drug administration: By precisely controlling the timing of MET inhibitor administration, the MET channel could be temporarily blocked during ototoxic drug treatment and restored afterward, minimizing the impact on hearing. 3. Local drug delivery: Using targeted drug delivery to directly administer MET inhibitors to the inner ear could reduce systemic side effects, protecting the inner ear from ototoxic drugs without affecting the MET channel in other parts of the body. 4. Dose control: Adjusting the dosage of MET inhibitors carefully may enable ototoxicity prevention while minimizing potential hearing damage. 5. Combination therapy: Using MET inhibitors in combination with other protective agents, such as antioxidants or anti-inflammatory drugs, could enhance their protective effect and reduce adverse impacts on hearing. In the current research field, many promising compounds have shown protective effects against ototoxicity in animal models, but translating these research findings into human clinical treatments still faces significant challenges. Animal models, especially those that mimic human diseases, provide valuable tools for understanding disease mechanisms and testing potential therapeutic methods. However, we must recognize that there are significant differences between animal models and humans in physiology, metabolism, and disease response, which may affect the efficacy and safety of compounds. Despite these challenges, we are optimistic about the progress of science and technology. With the development of precision medicine, we can more accurately identify and develop drugs targeting specific biomarkers and pathological pathways. In addition, the advancement of high-throughput screening technologies allows us to quickly assess the potential effects of a large number of compounds, while gene-editing technologies such as CRISPR/Cas9 provide new avenues for studying the role of specific genes in diseases. The development of these technologies is expected to accelerate the translation from the laboratory to the clinic. Addressing these questions will bring us closer to devising efficacious treatment modalities, prompting the realms of biology, pharmacology, structural biology, and clinical medicine to intensify their research and application endeavors in the arena of ototoxicity prevention and mitigation. As these scientific domains expand, we aspire to cultivate safer and more efficacious auditory protection strategies, mitigate hearing impairment caused by ototoxic medications, enhance the quality of life for patients, and catalyze further progress in the disciplines of otology and auditory preservation.

## Figures and Tables

**Table 1:** Drugs related to the MET channel and their mechanisms of action in protecting against ototoxicity

Compounds and their derivatives	Molecular structure diagram	Ototoxic drugs	Model system	The modes of actions and mechanisms of drugs in the field of otoprotection
UoS-7692 42, 43	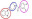	Aminoglycosides	Mouse cochlear culture; Zebrafish.	Blocking the MET channel, delaying the accumulation of aminoglycoside antibiotics in outer hair cells (OHCs), broad-spectrum protective effects, without affecting the efficacy of aminoglycoside antibiotics.
D-tubocurarine 44	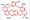	Aminoglycosides	Mouse cochlear culture; Zebrafish.	Blocking the MET channel, protecting outer hair cells, reducing the accumulation of aminoglycoside drugs within hair cells, exhibiting concentration-dependent protective effects, and showing ototoxicity at high concentrations.
Berbamine 44, 45	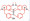	Aminoglycosides	Mouse cochlear culture; Zebrafish.	Blocking the MET channel, having a strong protective effect against aminoglycoside-induced hair cell death, and enhancing protective efficacy through structural modification.
FM1-43 31, 39, 48	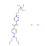	Aminoglycosides	Mouse cochlear culture; Zebrafish.	Blocking the MET channel, the fluorescent dye properties can be used to detect MET channel activity, and the protective effect is closely related to its chemical structure. Both the lipophilic tail and the positively charged head of the derivative are necessary for effectively blocking the MET channel, protecting hair cells, and chemical modification regulates the efficacy.
Carvedilol 35, 50	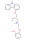	Aminoglycosides	Mouse cochlear culture; Zebrafish.	Blocking the MET channel, the fluorescent dye properties can be used to detect MET channel activity, and the protective effect is closely related to its chemical structure. Both the lipophilic tail and the positively charged head of the derivative are necessary for effectively blocking the MET channel, protecting hair cells, and chemical modification regulates the efficacy.
ORC-13661 54, 55	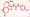	Aminoglycosides、cisplatin	Mouse cochlear culture; Zebrafish.	Blocking the MET channel, characterized by high affinity and permeability as a blocking agent, with membrane potential dependency, where the blocking effect is related to the cell's membrane potential. It protects hair cells from damage caused by aminoglycosides and cisplatin without affecting the efficacy of aminoglycoside antibiotics and cisplatin.
Phenoxybenzamine 56, 57	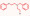	Aminoglycosides	Mouse cochlear culture; Zebrafish.	Reversible MET channel blockers, with a protective effect that is concentration-dependent, exhibiting ototoxicity at higher concentrations, and showing additive toxicity when combined with neomycin.
Benzamil 58	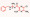	cisplatin	Mouse cochlear culture;	Blocking the MET channel, protecting hair cells from cisplatin-induced damage. Effective against cisplatin-induced damage in both *in vivo* and *in vitro* experiments.
